# Investigating the acceptance and use of massive open online courses (MOOCs) for health informatics education

**DOI:** 10.1186/s12909-023-04648-9

**Published:** 2023-09-08

**Authors:** Ali H Alharbi

**Affiliations:** Department of Health Informatics, College of Public Health and Health Informatics, Qassim University, Al Bukayriyah, Saudi Arabia

**Keywords:** Technology acceptance, E-learning, MOOCs, Health informatics

## Abstract

**Background:**

This study investigated the acceptance and use of massive open online courses (MOOCs) among health informatics educators and students in Saudi Arabian academic institutions. A theoretical model based on the unified theory of acceptance and use of technology (UTAUT), self-determination theory (SDT), and channel expansion theory (CET) was used to identify factors that affect MOOC adoption in health informatics education.

**Methods:**

A survey research design was employed, and cross-sectional data were collected from health informatics instructors and students in academic institutions in Saudi Arabia. A total of 145 completed responses were used in the final analysis of the data.

**Results:**

The findings indicated that performance and effort expectancy were important factors that could predict the acceptance and use of MOOCs among health informatics instructors and students. Additionally, perceived media richness affected the actual use of health informatics MOOCs among students and instructors in Saudi Arabian academic institutions. The results of this study show that autonomy, relatedness, and competence must be considered in the design of health informatics MOOCs.

**Conclusions:**

A combination of these models can effectively explain the adoption and use of MOOCs in emerging fields such as health informatics.

**Supplementary Information:**

The online version contains supplementary material available at 10.1186/s12909-023-04648-9.

## Introduction

Human life has been revolutionized by information and communication technologies (ICTs), which have had a significant impact on educational institutions. Digitalization has reshaped education by serving as a sociotechnical mechanism according to which both students and [1] teachers mutually take an interest in expanding and developing a digital model of education. Education has recently exhibited extensive growth in the context of online learning, or e-learning, following a shift in dissemination methods from traditional whiteboards to online learning platforms. Accordingly, a large number of courses are offered online by subject experts on platforms such as edX and MiriadaX [1, 2].

Kaplan and Haenlein [3] defined massive open online courses (MOOCs) as online courses that are accessible to anyone from any part of the world. Online courses are taught by experienced academics and subject experts who help students acquire the required knowledge and experience [4, 5]. MOOCs originated in Europe and the United States and have subsequently received worldwide attention, especially from distance education communities [6]. To embed MOOCs in higher education, top universities have begun to offer pedagogical-based learning objects to target a large number of students [1]. In this context, Stanford University introduced Coursera and Udacity, which allow academics to deliver valuable information to students, and Harvard and MIT began offering Harvardx and MITx, respectively, to more than 1.2 million students. In the Middle East, Jordan implemented the Edraak system to provide online courses to more than 12,000 students worldwide [6, 7].

One of the main reasons that learners are attracted to MOOCs is that MOOCs facilitate free online learning and flexible enrollment. MOOCs are easily accessible in terms of schedule, time, and location, and they benefit learners in distant geographical locations or students who have limited access to physical classroom education [8].

However, despite their advantages, MOOCs face challenges that hinder their acceptance, including noncompletion rates. Some students merely want to experience online learning instead of completing the courses; however, it is essential to maintain students’ continuous and consistent use of courses to ensure sustainability [9]. Therefore, it is important to investigate the factors that affect the adoption and acceptance of MOOCs globally [10].

In developing countries, according to Altalhi [6], the adoption rate of MOOCs is very low. This claim is especially true in Saudi Arabia. An increase in the growth and sustainability of MOOCs has been observed, and the literature has focused on this topic in developing countries; however, only limited studies have been reported in this context [6]. To the best of our knowledge, this study is the first to investigate this topic in Saudi Arabia by utilizing self-determination theory (SDT), channel expansion theory (CET), and the unified theory of acceptance and use of technology (UTAUT) to identify the factors that significantly influence the behavioral intention and actual use of health informatics MOOCs among educators and students in Saudi Arabian universities.

### Significance of the study

Investigating the adoption and use of MOOCs among educators and learners is essential to understand the extent to which this technology can reshape learning and teaching. Health informatics, as an emerging academic area, can benefit from the adoption of MOOCs to support teaching and learning. To design high-quality MOOCs to effectively support health informatics education, it is essential to understand the underlying factors and theoretical issues that might affect the adoption and use of this technology. An eclectic theoretical approach was used in the current study because no single theory is able to explain the relationships between the different factors that contribute to the acceptance and use of this technology, especially in emerging multidisciplinary areas such as health informatics. Therefore, the UTAUT, SDT and channel expansion theory were combined in this study to contribute to knowledge on the acceptance and use of health informatics MOOCs. These theories and models have the potential to explain the factors that affect MOOC adoption from different theoretical perspectives.

## Theoretical background

### Status of MOOCs in Saudi Arabia

Saudi Arabia has invested in new technologies to enhance students’ learning experience. Several academic institutions in Saudi Arabia have recently started to explore the potential of MOOCs to supplement traditional courses and prepare learners for the workforce. KKUx [11] is an initiative of the Deanship of E-Learning at King Khalid University (KKU) that provides high-quality digital content on the most important skills to prepare learners for their future jobs. Other platforms, such as Doroob and Rwaq, have also been developed to provide a range of open educational content that targets different audiences. Similarly, to boost the adoption of MOOCs in Saudi Arabia, another platform called Maarefh was introduced [12]. Recently, the Saudi national e-learning center also launched Future X [13], a national e-learning platform that supports the integration and delivery of diverse e-learning services and courses, including MOOCs.

### Health informatics MOOCs

Health informatics is an emerging field that has received considerable attention in recent years. Health informatics applies principles of computer science and information technology to the improvement of health care. Health informatics can be defined as “the interdisciplinary study of the design, development, adoption, and application of IT-based innovations in health care services delivery, management, and planning” [14]. Health informatics students and professionals should be knowledgeable about various technical, clinical, and administrative domains of health care.

To enhance health informatics education, it is essential for academic institutions and e-learning content providers to support the development of resources to fulfill the needs of learners in this emerging field. Few studies have discussed the role played by educational technology and platforms in supporting health informatics education. A learning management system (LMS) is central to any form of online and distance learning. Zakaria et al. [15] examined the use of a learning management system by 265 medical students and found that the students were interested in adopting various features of the LMS to enhance their learning.

Few studies have discussed the role and potential benefits of MOOCs in health informatics education. An exploratory study was conducted by Paton [16] on a website that offers a health informatics MOOC. The aim of this study was to investigate the extent to which this course was utilized by learners. The study revealed that more than 10,000 learners from more than 100 countries used the course between August 2012 and January 2014.

The success of a new system depends on users’ acceptance. Several studies have used the technology acceptance model (TAM) and the unified theory of acceptance and use of technology (UTAUT) to examine the acceptance of MOOCs in developed economies [1, 6, 17]. Fianu et al. [18] used the UTAUT model to identify the factors that affect the acceptance of MOOCs [19]. Although a considerable amount of research has been devoted to MOOC acceptance, less attention has been given to the adoption of health informatics MOOCs among learners and educators.

### Types of MOOCs and their characteristics

Although MOOCs were first introduced in 2008, there has been an extensive shift in the education sector from traditional learning to online learning in recent years, a trend that has been accelerated by the COVID-19 pandemic. There are two types of MOOCs: connectivity-based courses (cMOOCs) and instruction-based courses (xMOOCs) [19].

According to Shao [20], the most notable feature of MOOCs is their size. While a traditional e-learning system targets a limited number of learners, MOOCs are accessible by a large number of participants worldwide. MOOCs are also known for their openness and ability to support peer evaluation and reviews, which can enrich the learning experience. In addition, learners can easily access quizzes using modern devices [21].

### Challenges of adopting MOOCs in health informatics

MOOCs raise several challenges and issues as well as questions that have not yet been answered and need to be explored. A number of challenges to the adoption of MOOCs have been identified in the literature [22, 23]. Measuring learners’ performance and retention rate is one of the major challenges for MOOCs’ efficiency. A lack of interactivity and difficulty providing timely and relevant feedback for learners are among the most frequently cited factors that hinder learners’ engagement with MOOCs. Massive participation by a heterogeneous sample of learners with different cultures and backgrounds is also considered one of the greatest challenges in adopting MOOCs.

### The unified theory of acceptance and use of technology (UTAUT): an overview

The unified theory of acceptance and use of technology (UTAUT) model was developed and validated by Venkatesh et al. [24]. The UTAUT extends the traditional TAM by addressing social factors. It has been reported that the UTAUT has higher explanatory power than other models [6, 25, 26]. The UTAUT consists of four main constructs that predict a user’s behavioral intention toward and actual use of technology. These four constructs are performance expectancy (PE), effort expectancy (EE), social influence (SI), and facilitating condition (FC) [17, 27]. PE explains the degree to which users expect their use of a system to help improve their job performance, EE focuses on the ease of using a system, SI refers to the opinions of others regarding whether an individual should use a system, and FC refers to the support provided by organizational technical infrastructure with regard to using a system. System quality and user satisfaction (SQUS) as well as user-based use motives (UBUM) [17, 28] were added to the UTAUT. SQUS refers to the degree to which users are satisfied when using a new system. When the perception of a new system exceeds users’ expectations, users are satisfied [29]. The UTAUT model has also been extended by adding trust and flow experience [28]. Some studies have reported that the UTAUT can explain variation in behavioral intention (e.g., [6], [1], [17]). Previously, Venkatesh et al. [27] added four moderating variables to the UTAUT model: age, experience, gender, and voluntariness. According to [17], the UTAUT model has been cited more than 12,000 times. Although the model has been used extensively, the number of studies that investigate the acceptance of MOOCs remains limited, and the reported results have been mixed (e.g., [18], [30]).

### Self-determination theory (SDT)

SDT explains an individual’s intrinsic motivation in the absence of any external motivation, pressure, or influence. According to Deci and Ryan [31], intrinsic motivation occurs when an individual is engaged and interested in and begins to enjoy the task in question (in this case, using a new system). Nikou and Economides [32] defined intrinsic motivation as motivation resulting from a natural drive. SDT has been used extensively to study a variety of research topics, such as music education, video games, cloud-based virtual learning, social networking platforms, and health organization behavior [33–35]. SDT posits that three psychological determinants explain the “experience of choice”: relatedness, autonomy, and competence. Relatedness refers to caring for other people, connecting with peers, and seeking support from managers, colleagues, and family members when using a new system [36]. Autonomy pertains to the independence and freedom that the system offers to users to allow them to self-regulate their learning. Competence indicates that an individual accepts challenging tasks and has the confidence to achieve goals. Students’ belief in their ability to learn online via MOOCs and their belief that they have high levels of competence leads to higher motivation [37].

### Channel expansion theory (CET)

CET, which combines social presence with SI theories, media richness, and situational factors, suggests that media richness is the main factor that motivates an individual to adopt new technology and systems. This theory hypothesizes that individual experiences with regard to the development of knowledge affect channel richness. Such experiences, which consist of the organizational context, channel, communication partners, and messaging topics, allow users to learn about the new features, options, functions, and limitations of channels to ensure effective and efficient communication and elicit a perception of media richness. The influence of society on individuals during their use of media is an outcome of organizational context, which generates a new kind of knowledge. In addition, a better pattern of language and construction of messages can be experienced with a communication partner, while experience with a topic helps users employ specific terms and develop better communication skills, leading to improved media richness [38].

## Research model and hypothesis development

### Behavioral intention and media richness in the UTAUT

As noted in Sect. 2.5, the original model of the UTAUT includes PE, EE, SI, and FC. PE is similar to the perceived usefulness of the TAM; it explains how students can use MOOCs to improve their learning performance. EE is similar to the perceived ease of use in the TAM and describes the degree to which individuals believe that MOOCs are easy to use [39]. SI includes opinions and pressure from classmates, instructors, social groups, and friends regarding the use of MOOCs, which may increase students’ adoption tendencies and use of MOOCs.

Since MOOC platforms depend on multimedia technology, investigating the role played by media richness theory in the acceptance and adoption of MOOCs can provide greater insight into the factors that influence individuals’ intention to adopt MOOCs. Hew and Kadir [38] reported that media richness has a significant effect on behavioral intention. Rich media help users communicate more quickly and improve their performance. Media richness also supports users during e-learning. Therefore, we hypothesize that media richness affects learners’ perceptions of the possibility of using MOOCs and propose the following hypotheses:

#### H1

The behavioral intention to utilize health informatics MOOCs is influenced by performance expectancy.

#### H2

The behavioral intention to utili-ze health informatics MOOCs is influenced by effort expectancy.

#### H3

The behavioral intention to utilize health informatics MOOCs is influenced by perceived media richness.

#### H4

The actual adoption of health informatics MOOCs is influenced by perceived media richness.

### Behavioral intention and the actual use of MOOCs

A positive and significant relationship between behavioral intention and learners’ use of MOOCs has been reported [1]. According to [27], once learners are convinced to adopt a system, they are likely to do so. This relationship has been confirmed by [1] and [27]. Furthermore, behavioral intention mediates the relationship between SDT and the actual use of MOOCs [1]. Wan et al. [17] identified the mediating effect of behavioral intention and continued use intention. Previous studies have also reported the mediating role of behavioral intention in the relationships between self-determination, UTAUT, and task technology fit models and the intention to use MOOCs [10, 39]. We posit a positive relationship between behavioral intention and the actual use of MOOCs and thus propose the following hypothesis:

#### H5

Behavioral intention has a positive effect on the actual use of health informatics MOOCs.

### SDT, behavioral intention, and media richness

As noted in Sect. 2.6, the three attributes of SDT are autonomy, relatedness, and competence. Perceived relatedness may increase the motivation of users in a context featuring a supportive culture, a supportive environment and autonomy. Relatedness enhances belongingness and leads to a state of enjoyment for users of technologies and systems [34]. Learners are also influenced by people to whom they are connected, and relatedness creates bonds among learners or users in the workplace for mutual benefit. With regard to social and educational well-being, relatedness helps students investigate their behavior when using MOOCs [27]. Perceived relatedness is linked with autonomy, and it enables learners to make decisions regarding whether to use MOOCs. Previous studies have found a positive and significant relationship between perceived relatedness and the behavioral intention to use MOOCs [1, 38, 39].

Autonomy refers to users’ feeling that they have the freedom to adopt a new technology or system independently. With regard to the current study, autonomy indicates that students have the right to decide whether to use MOOCs and to enroll in any subject of their choice that is relevant to their field. MOOCs allow learners to choose their favorite subjects without limitations due to time, schedule, or boundaries. Several previous studies have found a correlation between perceived autonomy and behavioral intention (e.g., [40], [41]).

Perceived competence refers to individuals’ perception and belief that they are capable of accomplishing a specific task. High competence leads to a high level of motivation and encourages learners to investigate and attempt new things. Perceived competence can be affected by ease of use, language difficulty, connectivity, and digital skills. In e-learning environments, learners should be familiar with how to use digital platforms and interact via different communication channels, which is explained by CET [36, 38]. Based on the preceding discussion, we propose the following hypotheses:

#### H6

The behavioral intention to utilize health informatics MOOCs is influenced by perceived autonomy.

#### H7

The behavioral intention to utilize health informatics MOOCs is influenced by perceived relatedness.

#### H8

The behavioral intention to utilize health informatics MOOCs is influenced by perceived competence.

### Research methods

This study followed a quantitative cross-sectional approach, and data were collected via an online survey. A cross-sectional design is considered a time- and cost-effective approach. A structured questionnaire was adopted from previous studies to collect the primary data from the respondents. The questionnaire consisted of items related to constructs of the UTAUT, self-determination theory and channel expansion theory. All items were measured on a seven-point Likert scale. Details are given in Sect. 3.5. Prior to data collection, ethical approval was obtained for data collection. Informed consent was also obtained from the respondents. The aim of the study was explained to the respondents, who were informed that the data would be used only for academic purposes and that the identity of individuals and organizations would be kept confidential. Furthermore, the reputation of individuals and organizations would not be harmed. The respondents were given three to four days to complete the questionnaire. A total of 170 questionnaires were distributed to students and teachers, and 145 completed responses were received and used in the analysis.

### Sampling technique

The participants in the survey were faculty members and students from public and private universities in Saudi Arabia. The data collection took place from January to February 2022. A nonprobability convenience sampling technique was used to select the study sample. This sampling technique has been widely used in the social, management, and learning sciences [39, 42]. Related studies investigating MOOCs [1, 6, 17] have also used a convenience sampling technique to collect data. Prior to data collection, ethical approval was obtained from the scientific research ethical committee of Qassim University (Protocol # 21-14-11).

### Measures

To test the framework and hypotheses proposed in the current study, the first part of the questionnaire obtained the participants’ responses to items pertaining to each theory. The items were measured on a seven-point Likert scale ranging from 1 (strongly disagree) to 7 (strongly agree). The UTAUT comprises six constructs: PE (4 items), EE (4 items), SI (5 items), FC (5 items), behavioral intention (3 items), and actual use (4 items). SDT consists of three constructs, perceived relatedness (7 items), perceived autonomy (7 items), and perceived competence (6 items), which were adapted from [1] and [39]. Media richness (6 items) was adapted from [38]. The second part of the questionnaire focused on demographic information, such as gender, age, role as a teacher or student, sector, and level of education.

### Data analysis

The data were analyzed using structural equation modeling (SEM). Partial least squares SEM (PLS-SEM) was used to examine the reliability and validity of the scales. For this purpose, measurement models were developed and tested using PLS-SEM, and a structural model was developed and tested to test the hypotheses. The purpose of the measurement model was to evaluate the composite reliability, average variance extracted, and discriminant validity using heterotrait-monotrait (HTMT) ratios and Cronbach’s alpha coefficients [43]. The threshold criteria were as follows: composite reliability (CR) > 0.70, average variance extracted (AVE) > 0.50, Cronbach’s alpha > 0.70, factor loadings > 0.70, and HTMT ratios < 1. The structural model was tested using the bootstrapping method.

## Results

In total, 145 respondents participated in the online survey. Table 1 presents the participants’ demographic information. Among the respondents, 67.6% were male and 32.4% were female. In addition, 87.6% of the participants were students, and 12.4% were faculty members. Respondents mostly belonged to the 18–25 age group (86.2%), followed by the 26–35 age group (7.6%). Among the respondents, 90.3% were from public institutions, while 9.7% were from private institutions in Saudi Arabia. Additionally, 66.2% of respondents held bachelor’s degrees, 15.9% had a diploma, and 10.3% and 7.5% had master’s and doctoral degrees, respectively.

**Table 1:** Demographic information

Variable	Frequency	Percentage (%)
Gender		
Male	98	67.6
Female	47	32.4
Role		
Student	127	87.6
Teacher	18	12.4
Age		
18–25	125	86.2
26–35	11	7.6
36–45	7	4.8
46+	2	1.4
Institution		
Public	131	90.3
Private	14	9.7
Education		
Diploma	23	15.9
Bachelor’s	96	66.2
Master’s	15	10.3
Ph.D.	11	7.5

### Measurement model

A measurement model was developed to assess convergent and discriminant validity. Convergent validity was assessed by investigating factor loadings, composite reliability, average variance extracted, and Cronbach’s alpha coefficients. Confirmatory factor analysis (CFA) was conducted using PLS-SEM. Initially, Item 4 of PE, Item 3 of SI, Items 3, 5, 6, and 7 of perceived relatedness, Items 2, 3, and 7 of perceived autonomy, and Items 1, 5, and 6 of perceived competence were excluded from the analysis due to their low factor loadings. The criterion for such loadings, as noted by [43], is that they must be > 0.70. Furthermore, the criteria for reliability and validity are AVE > 0.50, Cronbach’s alpha > 0.70, and CR > 0.70, as suggested by [43]. Table 2 shows that the CR, AVE, and Cronbach’s alpha values met these thresholds, indicating that the scales used in the study were reliable and valid.

**Table 2:** Measurement model

Variables	Items	Loadings	CR	AVE	α
Performance Expectancy	PE1	0.905	0.882	0.715	0.800
	PE2	0.841			
	PE3	0.787			
	PE4	-			
Effort Expectancy	EE1	0.770	0.898	0.688	0.849
	EE2	0.854			
	EE3	0.855			
	EE4	0.836			
Social Influence	SI1	0.697	0.857	0.601	0.778
	SI2	0.771			
	SI3	-			
	SI4	0.821			
	SI5	0.805			
Facilitating Conditions	FC1	0.723	0.869	0.572	0.812
	FC2	0.769			
	FC3	0.782			
	FC4	0.808			
	FC5	0.693			
Perceived Relatedness	PR1	0.793			
	PR2	0.757	0.826	0.543	0.718
	PR3	-			
	PR4	0.679			
	PR5	-			
	PR6	-			
	PR7	-			
	PR8	0.713			
Perceived Autonomy	PA1	0.804			
	PA2	-			
	PA3	-			
	PA4	0.797	0.864	0.613	0.790
	PA5	0.735			
	PA6	0.794			
	PA7	-			
Perceived Competence	PC1	-			
	PC2	0.750			
	PC3	0.784	0.847	0.649	0.730
	PC4	0.878			
	PC5	-			
	PC6	-			
Perceived Media Richness	PMR1	0.729			
	PMR2	0.713			
	PMR3	0.727	0.889	0.574	0.851
	PMR4	0.736			
	PMR4	0.790			
	PMR5	0.842			
Behavioral Intention for MOOCs	BI1	0.861			
	BI2	0.838	0.885	0.719	0.805
	BI3	0.845			
Actual Use of MOOCs	AU1	0.784			
	AU2	0.792	0.856	0.598	0.776
	AU3	0.773			
	AU4	0.743			

CR: Composite reliability. AVE: Average variance extracted, α: Cronbach’s alpha

Discriminant validity was assessed using HTMT ratios, which is a robust statistical technique used to validate discriminant validity. HTMT ratios are used to determine whether the measures of a scale are related to each other. As shown in Table 3, sufficient discriminant validity was established, the scales used in the current study were found to be reliable and valid, and the measurement model had psychometrically sound properties.

**Table 3:** Discriminant validity (HTMT)

Variables	1	2	3	4	5	6	7	8	9	10
1.AU										
2.BI	0.909									
3.EE	0.581	0.655								
4.FC	0.67	0.858	0.925							
5.MR	0.815	0.897	0.604	0.767						
6.PA	0.681	0.81	0.733	0.837	0.752					
7.PC	0.702	0.897	0.667	0.835	0.86	0.826				
8.PE	0.625	0.745	0.892	0.909	0.597	0.693	0.64			
9.PR	0.631	0.702	0.758	0.856	0.698	0.739	0.723	0.644		
10.SI	0.676	0.893	0.795	0.882	0.8	0.805	0.828	0.89	0.71	

### Structural equation modeling

Bootstrapping was conducted to test the direct hypotheses. Standardized path coefficients, t-statistics, standard error values, bootstrapping lower and upper confidence intervals, and *p* values were calculated.

Table 4 presents the findings concerning the direct hypotheses. The UTAUT and SDT models explained 73.0% of the variance in the behavioral intention to adopt MOOCs. Moreover, the UTAUT and SDT explained 64.4% of the variance in perceived media richness. Further analysis indicated that behavioral intention and perceived media richness explained 55.8% of the variance in the actual use of MOOCs. These results are above the threshold > 0.35 [44], thus indicating large variances.

**Table 4:** Direct effects

Hypotheses	β	S.E	T Statistics (t > 1.96)	*P* Values	Support for Hypothesis
PE→BI	0.605	0.053	11.501	0.000	Yes
PMR→BI	0.503	0.062	8.078	0.000	Yes
BI→AU	0.465	0.096	4.854	0.000	Yes
PMR→AU	0.287	0.083	3.438	0.001	Yes
EE→BI	0.545	0.063	8.697	0.000	Yes
EE→AU	0.069	0.073	0.944	0.345	No
FC→BI	0.705	0.048	14.838	0.000	Yes
FC→AU	0.001	0.070	0.018	0.986	No
SI→BI	0.714	0.041	17.580	0.000	Yes
SI→AU	-0.049	0.087	0.558	0.577	No
PR→BI	0.540	0.078	6.883	0.000	Yes
PR→AU	0.068	0.097	0.699	0.485	No
PA→BI	0.655	0.063	10.432	0.000	Yes
PA→AU	0.049	0.075	0.651	0.515	No
PC→BI	0.691	0.046	14.889	0.000	Yes
PC→AU	-0.040	0.099	0.408	0.684	No

Table 4 shows that PE had a positive and direct significant effect on the behavioral intention to use MOOCs (β = 0.605, t = 11.501, p < 0.01). Further analysis of these results revealed that perceived media richness had a positive influence on behavioral intention to use MOOCs (β = 0.503, t = 8.078, p < 0.01) and the actual use of MOOCs (β = 0.287, t = 3.438, p < 0.01). In addition, EE had a significant and positive impact on behavioral intention (β = 0.545, t = 8.697, p < 0.01). Behavioral intention had a direct and positive effect on the actual use of MOOCs (β = 0.465, t = 4.854, p < 0.01). These results support H1, H2, H3, H4, and H5.

The results also indicated that SDT factors had positive effects on the behavioral intention to use health informatics MOOCs. Perceived relatedness had a positive impact on the behavioral intention to use MOOCs (β = 0.540, t = 6.883, p < 0.01). Perceived autonomy had a positive impact on the behavioral intention to use MOOCs (β = 0.655, t = 10.432, p < 0.01). Perceived competency had a positive impact on the behavioral intention to use MOOCs (β = 0.691, t = 14.889, p < 0.01). These results support H6, H7, and H8.

## Discussion

MOOCs have revolutionized teaching and learning. Traditional classroom teaching has been digitalized using ICTs, and MOOCs facilitate a new form of self-directed learning. MOOCs can help overcome the costs and accessibility issues associated with learning. The current study offers insights for future practice and research on health informatics MOOCs. The findings of the current study are consistent with those of previous studies on MOOCs [1, 6, 17, 39, 45]. The results of this study support the claim that the UTAUT model and SDT can explain the behavioral intention to adopt MOOCs. Furthermore, positive associations were found among behavioral intention, media richness, and the actual use of MOOCs.

The behavioral intention to adopt MOOCs is influenced by effort and performance expectancy. The intention to adopt MOOCs is also affected by SI. These findings may be due to the impact of and pressure from friends, family members, and classmates that encourage individuals to switch from traditional learning to online learning. The combined effects of the extended UTAUT model on behavioral intention are empirically consistent with the findings of [6] and [17]. The factors included in the UTAUT have a positive impact on media richness [36]. Flexibility, user friendliness, diversified methods of learning, interaction with professors, channels, organizational partners, and communication contribute to the media richness of MOOCs. The findings of the current study are consistent with those of previous studies [38, 39, 45].

Self-determination can explain the behavioral intention to adopt MOOCs. Perceived relatedness shows the preferences of learners to be connected with subject experts, perceived autonomy shows the importance of MOOCs with regard to supporting self-paced learning paths, and perceived competence is crucial for encouraging learners to adopt MOOCs. These findings are in line with [1].

Behavioral intention and media richness also have a positive impact on the actual use of MOOCs; these findings are in line with previous studies [1, 6, 17, 39]. MOOCs have the capability to offer learners diverse experiences via different communication channels.

The intrinsic motivation of individuals to use MOOCs can be driven by the three basic needs of relatedness, autonomy, and competence. The results of this study indicate that providing flexible and self-paced learning paths is essential for increasing the motivation of students to adopt health informatics MOOCs. MOOCs have received a great deal of attention from learners because they enable learners to engage with course modules at any time, the learning environment is pleasant and enjoyable, and there is no deadline for course completion. Students can reuse the lectures provided to them in video or audio form, and they can access the material from anywhere in the world.

The results of this study highlight the importance of media richness in health informatics MOOCs. It is essential for MOOCs to support the use of different channels to disseminate knowledge. Using animations, simulations, and other interactive features can enrich MOOCs and thus positively influence behavioral intention and the actual use of MOOCs among learners and educators in emerging fields, such as health informatics. For the non-supported hypotheses, the findings revealed that the convenience, usability and technical infrastructure of MOOC platforms do not directly enhance actual use; rather, they increase the behavioral intention to use MOOCs, which in turn enhances the actual use of MOOCs.

This study has a number of implications. The new model provides more robust and sophisticated findings that were overlooked in past studies. MOOC platforms need to support interactivity and enhance user interaction. Providing instant and constructive feedback is essential to enhance learners’ behavioral attention to and use of MOOCs. Integrating different aspects of multimedia into the design of MOOCs can encourage learners to adopt these courses. Another important factor that must be considered is the ability and flexibility of MOOCs to support learners’ autonomy and freedom to use and navigate MOOCs. Health informatics is a multidisciplinary area that attracts learners from different backgrounds. Therefore, it is recommended that a search engine or recommendation system be provided to support learners in finding MOOCs related to specific health informatics topics that are compatible with their preferences and needs and to help learners interact with people of similar interests and backgrounds.

## Limitations and future directions

Although this study contributes to the literature on the adoption of health informatics MOOCs among faculty members and students in Saudi Arabia, it has several limitations that should be taken into consideration when making generalizations. The study used cross-sectional data, and the sample size was relatively small. It is thus recommended that a mixed methods research design or longitudinal data be used to obtain a better understanding of the adoption of MOOCs within the health informatics education community. Future studies could use the task-technology fit model (TTF) and the notion of the technology user environment (TUE) in addition to channel expansion theory (CET) to investigate the adoption and use of health informatics MOOCs in Saudi Arabia. Future research could also investigate the moderating effects of other factors, such as self-regulation and personal readiness.

## Conclusion

Higher education institutions (HEIs) are becoming more interested in the possibility of adopting new models and approaches in the context of e-learning. MOOCs are gaining momentum and receiving increasing attention from the e-learning community in Saudi Arabia. The current study combined the UTAUT and SDT with CET to gain insights into the factors that affect users’ motivation to use MOOCs in health informatics education. The findings revealed that the UTAUT model can effectively predict users’ behavioral intention to adopt MOOCs and that the behavioral intention to adopt MOOCs and media richness influence the actual use of MOOCs. The results of the study support efforts to design and distribute MOOCs with the aim of supporting health informatics education. Health informatics MOOC providers should pay attention to integrating features into these courses to create more interactive learning environments.

**List of abbreviations**.

### Electronic supplementary material

Below is the link to the electronic supplementary material.

Supplementary Material 1

## Data Availability

The datasets used and analyzed during the current study are available from the corresponding author on reasonable request.
