# Supplementary material for: Investigating the acceptance and use of massive open online courses (MOOCs) for health informatics education
Source: BMC Med Educ. 2023 Sep 8;23:656. doi: 10.1186/s12909-023-04648-9 (PMC10492412; doi:10.1186/s12909-023-04648-9)
Supplement: Supplementary file 1 — Supplementary Material 1 [file 12909_2023_4648_MOESM1_ESM.docx]

**Appendix**

Appendix I Measurement Model PLS-SEM


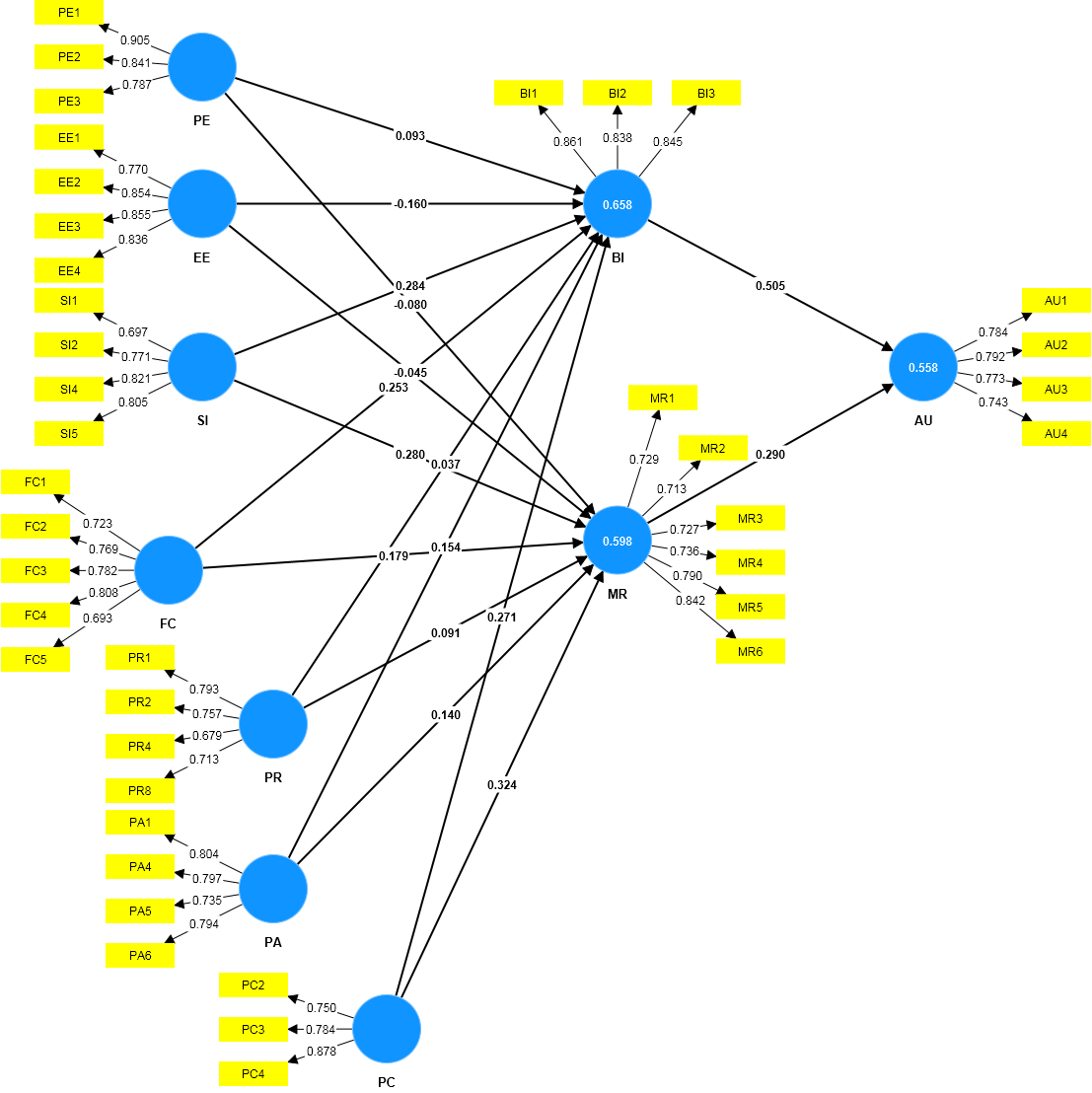


Appendix II Items for Each Variable/Construct

**SECTION A: Unified Theory of Acceptance and Use of Technology (UTAUT)**

| Strongly disagree | Disagree | Somewhat disagree | Neutral | Somewhat agree | Agree | Strongly agree |
| --- | --- | --- | --- | --- | --- | --- |
| 1 | 2 | 3 | 4 | 5 | 6 | 7 |

| **S#** | ***UTAUT* Items** | **References** |
| --- | --- | --- |
|  | ***Performance Expectancy*** |  |
| 1 | I would find MOOCs useful in my studies | Wan et al. (2020) [17] |
| 2 | Using MOOCs would help me solve problems in my studies |  |
| 3 | Using MOOCs would enable me to accomplish tasks more quickly |  |
|  | **Effort Expectancy** |  |
| 1 | My interaction with MOOC platforms is easy and understandable. |  |
| 2 | It is easy for me to learn how to use MOOCs. | Wan et al. (2020) [17] |
| 3 | I find MOOCs easy to use. |  |
| 4 | I have no difficulty in using MOOCs. |  |
|  | **Social Influence** |  |
| 1 | People who are important to me think that I should use MOOCs. |  |
| 2 | I find that using MOOCs is a fashionable and popular way to study in universities. | Wan et al. (2020) [17] |
| 3 | Professors/class fellows in my institution have been helpful in the use of MOOCs. |  |
| 4 | Using MOOCs makes me feel that I belong to the learning community. |  |
|  | **Facilitating Condition** |  |
| 1 | It is convenient for me to study in a MOOC platform. |  |
| 2 | I have the hardware necessary to use MOOCs. |  |
| 3 | I have the knowledge necessary to use MOOCs. | Wan et al. (2020) [17] |
| 4 | MOOCs are compatible with other learning resources I use. |  |
| 5 | Support from the platform is available when problems are encountered in MOOCs. |  |

**SECTION B: Self-Determination Theory**

***Instructions***: To what extent do you agree or disagree with the following statements on a 7-point scale:

| Strongly disagree | Disagree | Somewhat disagree | Neutral | Somewhat agree | Agree | Strongly agree |
| --- | --- | --- | --- | --- | --- | --- |
| 1 | 2 | 3 | 4 | 5 | 6 | 7 |

|  | **Perceived Relatedness** | References |
| --- | --- | --- |
| 1 | I truly like the people I work with. |  |
| 2 | I get along with people at work. | Khan et al. (2017) [1] |
| 3 | I consider the people I work with to be my friends. |  |
| 4 | People at work are pretty friendly toward me. |  |
|  | **Perceived Autonomy** |  |
| 1 | I feel like I can provide a lot of input in deciding how I use MOOCs in my teaching/learning. | Khan et al. (2017) [1] |
| 2 | When I am using MOOCs, I have to do what I am told. |  |
| 3 | My feelings toward MOOCs are taken into consideration at work. |  |
| 4 | I feel like I can pretty much use MOOCs as I want to at work. |  |
|  | **Perceived Competence** |  |
| 1 | I do not feel very competent when I use MOOCs in my teaching/learning. | Khan et al. (2017) [1] |
| 2 | My colleagues tell me I am good at using MOOCs in my teaching profession. |  |
| 3 | I have been able to learn interesting new skills in MOOCs through my profession. |  |
|  | **Section C: Channel Expansion Theory Media Richness** |  |
| 1 | MOOC features allow me to give and receive timely feedback. |  |
| 2 | MOOC features allow me to tailor my teaching/learning to my own personal requirements. |  |
| 3 | MOOC features allow me to communicate a variety of different cues (such as emotional tone, attitude, or formality). | Hew et al. (2016) [37] |
| 4 | MOOC features allow me to use rich and varied language in learning and teaching. |  |
| 5 | I could easily explain concepts using MOOC features. |  |
| 6 | MOOC features help me to communicate quickly and understand others. |  |
|  | **Section E: Behavioral Intention** |  |
| 1 | I intend to continue to use MOOCs for learning in the future. | Khan et al. (2017) [1] |
| 2 | I plan to use MOOCs for learning in the future. |  |
| 3 | I will insist on using MOOCs to study the courses I register for. |  |
|  | **Section F: Actual Use** |  |
| 1 | I often use MOOCs to manage my tasks. |  |
| 2 | I usually use MOOCs. | Altahi (2020) [6] |
| 3 | I regularly use MOOCs. |  |
| 4 | I frequently complete courses on a MOOC site. |  |
